# Reconciling imperatives: Clinical guidelines, antibiotic prescribing and the enactment of good care in lower-level health facilities in Tororo, Uganda

**DOI:** 10.1080/17441692.2022.2045619

**Published:** 2022-02-27

**Authors:** Susan Nayiga, Laurie Denyer Willis, Sarah G. Staedke, Clare I. R. Chandler

**Affiliations:** a Infectious Diseases Research Collaboration, Kampala, Uganda; b Department of Social Anthropology, The University of Edinburgh, Edinburgh, UK; c Department of Global Health and Development, London School of Hygiene & Tropical Medicine, London, UK; d Department of Clinical Research, London School of Hygiene & Tropical Medicine, London, UK

**Keywords:** Antibiotics, healthcare facilities, Uganda, stewardship, care

## Abstract

Faced with the threat of antimicrobial resistance, health workers are urged to reduce unnecessary prescription of antimicrobials. Clinical guidelines are expected to form the basis of prescribing decisions in practice. Emerging through evaluations of best practice – bundling clinical, technological and economic dimensions – guidelines also create benchmarks through which practice can be assessed with metrics. To understand the relationships between guidelines and practice in the prescribing and dispensing of antibiotics, ethnographic fieldwork was undertaken in lower-level health care facilities in rural Eastern Uganda for 10 months between January and October 2020, involving direct observations during and outside of clinics and interviews with staff. In a context of scarcity, where ‘care’ is characterised by delivery of medicines, and is constituted beyond algorithmic outputs, we observed that clinical practice was shaped by availability of resources, and professional and patient expectations, as much as by the clinical guidelines. For stewardship to care for patients as well as for medicines, a better understanding of clinical practice and expectations of care is required in relation to and beyond clinical guidelines.

## Introduction

It was getting late in the afternoon in Nagongera, Eastern Uganda. Awor, a midwife, was the ‘in charge’ for the day, and running the three-roomed health centre alone. While in the consultation room she obtained the clinical history of patients, wrote diagnoses, and completed the outpatient register. She then went to the laboratory to conduct rapid diagnostic tests and fill out the laboratory register. From there, she moved to the dispensing room to give patients the medicines prescribed. Her colleague Aketch, a nurse, was carrying out an immunisation outreach in the neighbouring village. Awor had seen 9 patients since opening the doors that morning and there were over 10 patients waiting to see her still. A 19-year-old woman entered the consultation room with an almost inaudible greeting to which Awor responded briefly and reached out for the patient book. She then looked at the woman who was seated on a chair facing her and readied herself to listen to her health complaints. The woman explained that she had a persistent headache, felt pain when passing urine and had pain in the stomach. Awor asked her to indicate on her body where she felt the pain. The woman touched the lower part of her abdomen. Without asking further questions, Awor wrote in the patient’s record book. She diagnosed the woman with a urinary tract infection and wrote a prescription for her including two antibiotics – nitrofurantoin and doxycycline – and paracetamol. The current clinical guidelines specified Awor should carry out a urine analysis – using a urine dipstick test and microscopy which were never available at the health centre – and if positive prescribe nitrofurantoin, but not usually doxycycline. She told the patient to sit in the waiting area next to the dispensing window and turned to explain the case to SN, saying that it was common for adolescent girls to contract urinary tract infections. She flipped the pages in the patient’s record book, reading through to understand her past medical history. She said that on her last visit at one of the government health centres in the area, the woman had been diagnosed with a urinary tract infection and prescribed doxycycline. Awor wondered if the urinary tract infection was not responding to treatment and she considered changing the antibiotic prescription to another antibiotic, levofloxacin, which Awor considered effective for treating persistent infections. However, because levofloxacin was not available at the health centre Awor worried that it might not be affordable at the local pharmacies. Awor continued to read through the previous treatment that the woman had received and then she told me (SN) that the problem might be taking frequent self-treatment with antibiotics adding that ‘with adolescent girls, you can never know’. Awor then called the woman back to the consultation room and asked her to explain how she had taken the last treatment with doxycycline. The woman explained that she had taken it every 6 hours. Awor told her that she was supposed to take it every 12 hours. She then decided to change the prescription to ciprofloxacin, the second line treatment, which was available at the health centre. She believed ciprofloxacin was a strong antibiotic that would be effective for this woman’s infection. Throughout this encounter, Awor did not directly mention drug resistance as the cause for needing a stronger antibiotic treatment although this was implied by her concern that the patient had taken repeated courses of antibiotics.

At a health centre used to routine stockouts, more patients than time or health workers can adequately address, and a high prevalence of recurrent infection in the region, the case above reflects the complexity of providing ‘good care’ in a primary health care facility in a context of scarcity in rural East Africa where this research took place. Increasingly, however, health workers like Awor have been tasked with juggling even more complexity within this kind of routine encounter: pressure is mounting to make and amend their decisions around treatment and care within the guidelines laid out by antimicrobial stewardship programmes that aim to achieve the goal of optimising antimicrobial use in healthcare settings.

Antimicrobial resistance (AMR) is an increasing public health threat worldwide, which is understood to be driven by the widespread use of antimicrobials. High levels of antibiotic use have been reported in some low- and middle-income countries (LMICs) although country level data is limited (World Health Organisation, 2021). To minimise the spread of AMR, global health actors and policy makers have emphasised the need to reduce the use of antibiotics, but this is a challenging task (Cañada, 2021). The World Health Organisation’s Global Action Plan (GAP) on AMR highlights the need for antimicrobial stewardship to ensure that antimicrobials remain effective for use by current and future generations (World Health Organisation, 2015). Stewardship interventions are envisioned to reach prescribers, dispensers, patients, policymakers and the general public. So far, stewardship programmes in hospitals have shown some success in high income (Davey et al., 2017) and low-income country settings (Akpan et al., 2020; D’Arcy et al., 2021; Kerr et al., 2021) but few have been piloted in LMICs, particularly in outpatient treatment settings (Wilkinson et al., 2018).

Clinical guidelines provide a platform through which global and national actors can govern care; they emerge through evaluations of best practice – bundling clinical, technological and economic dimensions – and they create metrics through which to assess practice (Dixon & Chandler, 2019). On the global health stage, guidelines become a device through which to parse complexities of care across multiple contexts; by altering the algorithm of a guideline in one setting, the number of antibiotic prescriptions could be reduced in another. Prescribing practice can be benchmarked against the latest guidelines which is a key tenet of antimicrobial stewardship programmes. Guidelines connect places and people, through paper, technology or networks of learning, and can be understood as an actor, part of the assemblage that creates cases, epidemiology, material flows and ultimately shape care (Tompson & Chandler, 2021). However, the practice at the end of a guideline’s journey is not solely informed by the parameters of the written algorithm. Clinical practice must be understood as situated, contingent upon patterns of resources, professional and patient expectations, and algorithms that guide care (Beisel et al., 2016; Chandler et al., 2008; Street, 2014). For stewardship to care for patients as well as medicines, a better understanding is required of clinical practice and how guidelines translate in different settings.

In this article, we open up the tension between an emphasis on clinical guidelines and the wider remit of health workers to provide care. Practices that we see as part of care are best understood within their context. Aryn et al. (2015), for example, write that ‘what care looks and feels like is both context-specific and perspective-dependent’ (Aryn et al., 2015, p. 1). Focusing on the way care is brought into being, Mol (2008) argues that ‘good care is something that grows out of collaborative and continuing attempts to attune knowledge and technologies to diseased bodies and complex lives’ (Mol, 2008, p. 2). Building on Aryn and Mol’s work, we consider how conditions of scarcity adapt what care becomes, in a context that has been on the continuous receiving end of transnational intervention that has reinforced a pharmaceuticalised form of case management (Dixon & Chandler, 2019). In this way, this paper provides new considerations for antimicrobial stewardship programmes: it demonstrates how clinical guidelines co-exist with other strategies to achieve care in settings where resources are limited.

### Action on antimicrobial resistance in Uganda

Following the initiation of the World Health Organisation (WHO) Global Action Plan on AMR in 2015, outlining mechanisms for containing AMR, a country assessment of efforts to curb AMR was conducted by the Global Health Security Agenda in 2015 revealing that there were no coordinated efforts in Uganda to address AMR (Uganda National Academy of Sciences, 2015). Aiming to develop an action plan for AMR to draw stakeholders together, a situational analysis of AMR in Uganda supported by the Uganda National Academy of Sciences (UNAS) and the Center for Disease Dynamic, Economic and Policy (CDDEP) under the Global Antibiotic Resistance Partnership (GARP)-Uganda revealed increasing trends in AMR (Uganda National Academy of Sciences, 2015). The situational analysis that was conducted in 2015, following a blueprint used in multiple LMICs, focused on quantification of gaps – between what people should do and know, and what was captured in practice. This included a particular focus on the size of inappropriate treatment and the gap in knowledge and practice. The report also highlighted limited awareness among the public, policy makers, prescribers and other professionals about AMR and its consequences.

The AMR situational analysis and recommendations made by UNAS in 2015 informed a number of revisions focusing on antimicrobials in the 2016 clinical guidelines. The Appropriate Medicines Use Unit housed in the Health Services, Pharmacy department of the Ministry of Health made revisions leading to the 2016 clinical guidelines through a six-month process involving consultations with public health programme staff, medical experts and health workers of all cadres guided by scientific evidence, expert knowledge of the Ministry of Health and the WHO considering socio-cultural factors, health system resources and cost effectiveness of resources (Uganda Ministry of Health, 2016b, 2017). The 2016 guidelines stressed the need to ensure high standards of quality and efficiency in service delivery, an efficient medicines and health supplies procurement and supply system and efficient use of limited resources to improve rational prescribing (Uganda Ministry of Health, 2016b). The 2016 Essential Medicines and Health Supplies List for Uganda was harmonised with the clinical guidelines of 2016 to reflect medicines and diagnostic requirements for management of common conditions classified by the ‘level of care’ corresponding to the services available at those levels, and also by the vital, essential and necessary classification (Uganda Ministry of Health, 2016a). Operating under a rubric of rational drug use as promoted by the WHO since 1985 as defined at a conference of experts in Nairobi (World Health Organisation, 1985), the 2016 guidelines emphasise the need to promote *access*, clinical *effectiveness* and appropriate use of *resources* to prevent negative consequences such as antimicrobial resistance. The guidelines also include a section on AMR highlighting its drivers and listing actions that can be taken by health workers and patients to curb the problem.

In 2018, Uganda’s National Action Plan (NAP) on AMR was launched by the Ministries of Health, Agriculture, Animal industry and Fisheries and Water and Environment, emphasising the need to promote the prudent use of antimicrobial agents. The NAP summarised activities to characterise and situate the AMR problem in Uganda, guided by numerous international actors and frameworks. The NAP includes a strategic objective to optimise the use of antimicrobial drugs through effective stewardship practices (Government of Uganda, 2018). Achieving optimal antimicrobial use is understood to require ‘strengthening technical and regulatory frameworks, ensuring availability of appropriate medicines and changing behaviour among prescribers, dispensers and consumers’. Antimicrobial stewardship programmes target prescriber behaviour change through interventions designed to promote the appropriate prescription of antibiotics. Antimicrobial stewardship interventions consist of planned activities, including (1) regularly updating and ensuring availability of guidelines, (2) supporting proper functioning of medicines and therapeutics committees in all health care facilities, and (3) disseminating antimicrobial stewardship working manuals. These activities are coordinated by a Technical Working Committee on Antimicrobial Stewardship, Optimal access and use of antimicrobials constituting officials from the Ministries of Health, Agriculture Animal industry and Fisheries and Water and Environment and other partners led by the Ministry of Health, Health Services, Pharmacy department.

## Methods

Ethnographic fieldwork took place between 22 January 2020 and 26 February 2020 and 5 October 2020 and 29 October 2020 in Tororo district. The ethnographic research was conducted with healthcare providers from two government Health Centre (HC) IIs. HCIIs are the entry point of the multi-layered public health system in Uganda with HCIIs at parish level, HCIIIs at sub county level, HCIVs at county level, district hospitals, regional referral hospitals and a national referral hospital. An ethnographic approach aiming to capture the context of prescribing and dispensing antibiotics as part of our Antimicrobials In Society research project (Antimicrobials In Society Hub, 2017) was considered most appropriate for exploring the lived realities of health care provision in rural health centres. The ethnographic research was led by SN, a Uganda Social Scientist that had conducted in-depth ethnographic fieldwork in households located in the catchment area of the two-level public health facilities where this research was conducted. SN carried out fieldwork over eight weeks including five weeks of participant observations in one health centre (HCA) and three weeks in a second (HCB). She stayed at the health centres for 5–7 hours, three days a week. Written consent for participant observations and interviews was obtained from health facility staff at recruitment. During her time at the health facility, SN positioned herself in the consultation room assisting with simple administrative tasks such as recording patient information in the outpatient department register. This enabled her to observe interactions between health workers and patients during consultations, witnessing the process of diagnosing and treating patients. Between May and June 2020, fieldwork was restricted due to the COVID-19 pandemic, so follow-up interviews with health workers were conducted remotely by phone.

### Study setting

Tororo is a rural district located near the Kenyan boarder, with an estimated population of 517,080 residents living in 102,492 households (Uganda Bureau of Statistics, 2017). Tororo is an agricultural community, with over 70% of the population involved in subsistence farming (Tororo District Local Government, 2015). Poverty is common in Tororo, with over 50% of the population living on less than 1 USD per day (ibid). Tororo has 65 government health facilities, but most lack equipment, electricity, running water and essential health workers and suffer frequent stock outs of supplies (Medicines and health service delivery monitoring unit, 2014). In Tororo, there is one doctor for every 43,144 residents, compared to one doctor for every 20,000 residents at the national level (Tororo District Local Government, 2015). Following decentralisation of Uganda health systems in 1993/1994 (Gonzaga et al., 1999), NGOs and the private sector proliferated, playing a larger role in the provision of health care (ibid). Most local residents rely on the private sector, including drug shops and clinics (Nayiga et al., 2020).

### Primary health care in Tororo

The health centres in this study were out-patient clinics that managed common diseases affecting local residents, such as malaria and offered antenatal care. HCA, which had opened in September 2019, was located in a five-roomed building. The health facility was run by a midwife who normally shared roles with one nurse. HCB had been in operation for over five years and was run by a nurse, a nursing assistant and a volunteer. HCB was located in a three-roomed house and thus space for work was limited. All the work was done in one room. On average about 25 patients were seen daily at both health centres except following the National Medical Stores medicines delivery. On those days, up to 40 patients might present.

HCIIs have limited laboratory capacity and basic equipment. At HCA, only malaria rapid diagnostic tests (RDTs) could be done while at HCB RDTs were available for HIV, syphilis and malaria. HCA had a weighing scale and a blood pressure machine but neither piece of equipment was functioning six months later because the batteries had not been replaced. At HCB, the digital weighing scale was old and faulty and there was no blood pressure machine. Both health centres did not have thermometers, running water and electricity. The National Medical Stores supplied HCIIs a limited range of medicines that were considered ‘essential’ at this level of care. For instance, antibiotics supplied included amoxicillin, nitrofurantoin, metronidazole, doxycycline, and ciprofloxacin. Stock outs of antibiotics were common at the health centres.

In both health centres, one book copy of the Uganda Clinical Guidelines 2012 (National guidelines for management of common conditions) was available and kept on the table in the consultation room. Notably, the latest version of the clinical guidelines - Uganda Clinical Guidelines (UCG) 2016 had not yet been supplied to the health centres when we carried out this fieldwork. The guidelines were perceived by health workers as the yardstick for measuring health worker performance when it came to patient management and use of medicines. Interestingly, recognition by health workers of the underlying values of the guidelines contrasted the way the guidelines were used in practice. In the global health community, AMR is currently framed as a problem arising from irrational use of antimicrobials by health care providers who are said to be injudiciously prescribing it. Understanding the context of antibiotic prescribing brings to the fore, how narrow and removed from the realities of healthcare settings in Uganda the current focus on ‘rational use’ of antibiotics is, particularly in settings characterised by scarcity of resources. We observed that clinical practice in lower-level health facilities in Nagongera, Tororo was shaped by patterns of resources, professional and patient expectations as much as by the guidelines as we explain further in the next sections.

## Results

We found that clinical guidelines were present, and known in the health facilities, but seemed to co-exist with clinical practice rather than dictate it. In reality, health workers would go days without consulting the guidelines except for when they were faced with complicated, rare or confusing cases. In the first part of this section, we focus on scenarios where the health workers consulted the clinical guidelines but did not find them helpful. Then, we describe how health workers negotiated resource constraints at the health centre. Lastly, we describe efforts of health workers to cover possible infection using antibiotics.

### Shortcomings of the clinical guidelines in everyday practice at the health centre

You know changing giving of medicine, it becomes a bit tricky because we always use the same guideline unless that guideline changes, that is when you change the way of giving medicine. So you need to follow that guideline and it is the book we always use. I even showed you my bible (referring to the clinical guidelines) there.

Obbo, a nurse in HCB, explained his view of the guidelines to SN when they discussed ongoing efforts to address what was described as ‘inappropriate’ prescription of medicines by health workers. Obbo was a nursing assistant in his late forties and had served as a health worker in three government health centres in the local area for close to twenty years. Being originally from the area, Obbo personally knew most of the patients that presented at the health centre and was very popular. Obbo was also the chairman of the parent teacher’s association of one of the secondary schools in the local area. Faced with challenges of lack of accommodation at the health centre and high costs of transport to get there, the health workers at HCB had agreed on a schedule of who worked on what days. On one Monday morning, Obbo was working alone at the health centre. A 13-year-old boy presented with a headache and difficulty swallowing food because of pain in the throat. Obbo consulted the clinical guidelines but after a while flipping through the pages he closed the book and said ‘some conditions are not included in the guidelines’. He then decided to classify the boy’s condition as tonsilitis, and he said that he would prescribe what he would have prescribed for cough adding that ‘the easy thing for us here is amoxicillin’. He instructed the boy to go to a drug shop and buy amoxicillin because it was out of stock at the health centre.

Amoxicillin was described by health workers as the penicillin drug of choice at HCIIs. ‘We are supplied 8 tins of amoxicillin to last two months but it gets used up in one and a half months and then we ask patients to buy it’, Aketch a nurse at HCA said as she explained that amoxicillin was used to treat so many conditions at the health centre. Aketch, a vibrant woman in her early thirties had been recently recruited. She had previously worked in a private hospital in the neighbouring district. She said that she had always aspired to work in a government health facility because of the job security and the opportunities for further studies that the job offered. She made plans to enrol for a degree in nursing after two years of serving at the health centre. She also planned to take a bank loan and invest in land. One afternoon, Aketch treated a neonate that presented with a fever and cough. Aketch examined the child’s umbilical cord and said it had broken off leaving a wound that looked like it was being poorly managed. She consulted her colleague Awor, a midwife in charge of the HCII, to help with the management of the neonate. Awor was a reserved and calm woman in her fifties and had been a health worker for over 15 years. Awor had worked in a hospital in Northern Uganda for several years and in a HCIII and HCII in the local area before joining HCA. On examining the baby’s umbilical cord, Awor suggested that they prescribe ampicillin syrup but wondered whether the mother would be able to find it in the local drug shops. Puzzled about the cause of the fever and cough, the health workers turned to the clinical guidelines section on neonatal infections. ‘This book is too brief and summarised’, Aketch said, explaining that they were not able to find the guidance they needed for this case. They diagnosed the neonate with bacterial infection with abdominal colic and asked the caretaker to buy ampicillin syrup as it was not available at the health facility.

These scenarios reflect some of the challenges that health workers faced on the few occasions that we observed them consult the guidelines. The guidelines were sometimes found to be brief, vague and not exhaustive for some conditions. At the same time, providing care meant that health care workers had to make adjustments to suit the actual availability of antibiotics in the clinic, the ability of patients to pay (or not) for other antibiotics elsewhere, and whether private pharmacies would have an antibiotic available too.

### Negotiating resource constraints at the health centre

We observed that nurses’ conclusions on possible bacterial infections seemed to be based on patient symptoms. This was the case when Aketch managed a 20-year-old woman that presented with a discharge, sores and itching of her private parts as well as lower abdominal pain. Aketch explained that based on these symptoms, she would have wanted to test this woman’s urine and blood. She suspected that the woman had candida, a urinary tract infection or syphilis and so she prescribed metronidazole, doxycycline, clotrimazole cream, ibuprofen and nitrofurantoin. Aketch said that usually patients ended up leaving the health facility with three or four antibiotics because treatment was presumptive. She explained that each antibiotic she prescribed targeted a specific problem and system in the body. What we see here is an effort on Aketch’s part to provide good care for infections in a context of scarcity, providing insights into some of the reasons behind health worker prescription practices.

Nurses were faced with cases that were complex and did not fit neatly within the guidelines. They often complained about having to make diagnoses which they considered to be beyond their scope of training. They explained to SN that clinicians and medical officers would be expected to do more investigations and give more precise diagnoses. However, Ugandan clinicians can be found in HCIIIs in the Ugandan public health system while medical doctors are stationed in health centre IVs and district hospitals. Nurses often gave broad diagnoses allowing them to prescribe the antibiotics that were available at the health centre as Awor explained. She said;

It is hard to diagnose these conditions. That is the reason we end up going with broad diagnoses like acute respiratory tract infection (ARTI). ARTI could include common cold, bronchitis, TB and many others. Because we are not sure what the actual ARTI is, and we have no capacity to test we go with the broad category and it is known that this class of diseases is treated with antibiotics and so we prescribe the antibiotics.

Persistence of patient symptoms following treatment with an antibiotic was sometimes interpreted by nurses to be an indicator of a possible bacterial infection. One morning at the health centre, a woman came into the consultation room with an 8- month-old baby boy. She explained that he was having difficulty breathing and that his nose was blocked. Awor observed the movement of the baby’s chest and said his breathing was fine. She felt the baby’s forehead using the back of her hand and said the body was not hot. Nonetheless, she sent them to the next room for a malaria RDT which was negative. Awor wrote the diagnosis of bacterial infection and asked the mother if she could afford to buy erythromycin syrup for the child and the mother said yes. Awor told SN that two weeks ago this baby was brought to the health facility with a cough and she prescribed amoxicillin. Now that the mother had brought him back with the same complaint, she suspected that the cough was bacterial but was not covered by amoxicillin so she prescribed erythromycin, which she considered to be a strong antibiotic. She said that if the cough persisted after the child has been given erythromycin, then she would opt for stronger antibiotics such as azithromycin and cefalexin.

### Antibiotics to cover possible infection

Health workers occasionally prescribed antibiotics that would be described as ‘inappropriate’ if they anticipated delays in following recommendations for referral to a higher-level health centre. Health workers explained that patients many times did not follow through with referrals because of concerns that they would get admitted or find large numbers of patients and sometimes not even find drugs there. Aketch explained that;

When a patient presents with certain complaints that you feel require further investigation and you need to refer to the HCIII, you cannot just let them go with no medicine. You have to give them something for the first five days as they organise to go to the health centre and usually this includes an antibiotic.

This was the case for a 70-year-old man that presented at the health centre with pain in his hand and the joints of his shoulder, swollen feet and running short of breath. On measuring the patient’s blood pressure, Aketch said it was low, as was his pulse. Aketch said that the symptoms this man had, pointed to a problem with the heart. Awor said that they needed to prescribe an antibiotic because they did not know the root cause of these problems. She said they needed to give a strong antibiotic like ciprofloxacin because the old man could have had tuberculosis. The old man was diagnosed with hypotension, and injury to the shoulder joint and prescribed ciprofloxacin, dexamethasone, fefan and vitamin B complex. They referred the patient to the HCIV for further management. Anticipatory use of antibiotics was also observed where possible infection was perceived by the health worker. One day, Awor managed a case of two girls that she classified as having scabies. She prescribed benzyl benzoate solution but also decided to give amoxicillin in addition for possible infection as she explained saying: ‘The amoxicillin in this case is to manage the possible infection from the pus that comes with the scabies skin infection that may spread in the blood stream if not managed’. At the core of the nurses’ everyday practice was the desire to give good care. Caring for patients in this setting required going beyond the framework of the guidelines to accommodate the health facility capacity and the social context.

## Discussion

Clinical guidelines are a fundamental tool that antimicrobial stewardship programmes rely on to guide, change – and assess – clinical practice. Introduction of new medicines, changes in international and local policies, technologies, diagnostic tests and diseases inform revisions of clinical guidelines by experts at the national level with the expectation that this will translate into changes in clinical practice across multiple health care settings. Our ethnographic observations and interviews with health workers in lower-level public health facilities contribute a fine-grained analysis of the context of prescribing and dispensing antibiotics in the presence of guidelines in the everyday life of the clinic. Our findings reveal that clinical practice is shaped by patterns of resources, health worker training and patient expectations supported by information provided in the clinical guidelines. In a context of scarcity, antibiotics are vehicles of care enabling health workers to cope with gaps in laboratory capacity and health worker training as well as managing possible infection. Although antimicrobial stewardship programmes emphasise protection of medicines to curb resistance, in practice health workers in lower-level health facilities are focused on prescribing antibiotics available in their limited arsenal, hoping to treat possible resistant infections, cover possible infection, and provide prophylaxis to prevent bacterial infections from developing. For antimicrobial stewardship programmes to protect medicines as well as care for patients, clinical practice must be understood both in relation to and beyond the framework of the clinical guidelines.

The implementation of clinical guidelines intended to establish a strong foundation for the appropriate and efficient use of medicines by restricting these commodities is a potential challenge for front-line workers in low-income settings where health care has often been reduced to the provision of medicines (Dixon & Chandler, 2019). The focus on antimicrobial stewardship amidst increasing concerns about AMR conflicts with the notion of good care built around medicines in low-income settings as it emphasises protection of medicines by restriction (MacPherson et al., 2021). The evidence from this research calls for the widening of the focus of antimicrobial stewardship beyond prescriber practices so as to understand the potential harm of emphasising the use of clinical guidelines in a context of scarcity. Antimicrobial stewardship needs to address care itself by returning to the basics that emphasise care without medicines such as counselling and support of patients but also the inequities written into the health system (Denyer Willis & Chandler, 2019). In addition, we found that in the absence of latest clinical guidelines of 2016 in the HCIIs, health workers relied on the 2012 guidelines, which only briefly described some of the topics. Expanding the guidelines to include more information on the differential diagnosis would encourage health workers to think beyond antibiotics. The absence of updated guidelines is a gap that needs to be addressed in lower-level health facilities if clinical practice is to be impacted by the revision of clinical guidelines.

Health worker decisions sometimes conflicted with the values of ‘appropriate medicine use’ underlying the clinical guidelines as they turned to antibiotics as quick fixes for care in a broken health system (Denyer Willis & Chandler, 2019). Beyond the framing of the problem of irrational use of medicines among health workers as rooted in the lack of knowledge about optimal use of antimicrobials (Kamuhabwa & Silumbe, 2013), what we observed was health workers tailoring their use of the clinical guidelines (or not) to accommodate their limited training and the lack of resources in their setting as has been observed elsewhere in the use of medicines and technologies (Boonen et al., 2017; Tarrant et al., 2021, 2020). Optimising antibiotic use calls for recognition that clinical guidelines are not received in a vacuum and as such clinical practice is shaped by patterns of resources, health worker training and patient expectations (Broom et al., 2020; Nabirye et al., 2021; Yantzi et al., 2019) as much as the guidelines. In addition, understanding the logic behind health worker prescription decisions would be key in designing locally relevant stewardship intervention packages.

In our research setting, we found that health workers suspected resistance when a patient seemed to have failed on an antibiotic informing their decision to prescribe what they considered to be a stronger antibiotic. The practice of recognising resistance through treatment failure and turning to stronger antibiotics has also been reported in studies conducted in other LMICs (Cañada, 2021; Pearson & Chandler, 2019). The continued focus on resistance by stewardship programmes may have an unintended consequence of driving unnecessary use of antibiotics if not addressed (Pearson & Chandler, 2019). In addition, other scenarios such as the management of infections with bacteria that were not covered by the originally prescribed antibiotic and management of non-bacterial infections as well as non-infectious causes of infection would have to be addressed as part of stewardship intervention packages.

The stories presented in this paper contribute to a growing body of critical global health scholarship that highlights how global health initiatives impact care in ways that may not be beneficial to the local populations in resource-constrained contexts (Biehl, 2016; McKay, 2018; Whyte et al., 2013). Health interventions implemented with a narrow focus of addressing one or two health problems often have had the unintended consequence of creating a situation of ‘abundance and scarcity’ that undermines local healthcare systems (Brown, 2015; Prince & Otieno, 2014). Dominance of the global north in shaping the global health agenda and policies, as we see with AMR, is a reflection of the way that global health continues to operate as an apparatus of coloniality enabling and maintaining global health inequities (Anderson, 2014; Biehl, 2016; Richardson, 2020). Some elements of global AMR policies such as following a guideline and relying on laboratory diagnoses to guide antibiotic prescription have been shown to be ‘non-scalable’ in low resource-contexts where laboratories are very few (Cañada, 2021). In a context of scarcity, clinical practice is tailored to accommodate deficiencies in the health care system (MacPherson et al., 2021; Nabirye et al., 2021; Prince & Otieno, 2014), and so health worker practices need to be understood beyond a rubric of ‘noncompliance’. Disseminating clinical guidelines alone is not enough to see a reduction in antibiotic use in healthcare settings. Beyond disseminating guidelines, antimicrobial stewardship programmes will also require investment in the health infrastructure to improve health workers’ ability to deliver quality care beyond the provision of medicines.
